# Single‐cell DNA methylation profiling: Technologies, computation, and applications in precision oncology

**DOI:** 10.1002/1878-0261.70303

**Published:** 2026-07-10

**Authors:** Ik Soo Kim

**Affiliations:** ^1^ Department of Microbiology Gachon University College of Medicine Incheon South Korea

**Keywords:** clinical epigenomics, epigenetic therapy, intratumoral heterogeneity, multi‐omics, single‐cell methylomics

## Abstract

Cancer is an intrinsically heterogeneous disease characterized by distinct malignant subclones defined by specific genetic and epigenetic alterations, such as aberrant DNA methylation. Traditional bulk sequencing methods analyze large populations of cells in aggregate, yielding an averaged methylome signal that masks the rare but clinically significant epigenetic patterns driving tumor initiation, metastasis, and therapeutic resistance. The emergence of single‐cell DNA methylation (scDNAme) sequencing has driven a paradigm shift in oncology by providing the resolution required to dissect this intratumoral heterogeneity. By profiling the epigenome of individual cells, scDNAme analysis enables the discovery of novel aberrant patterns, the precise reconstruction of cellular lineages, and the characterization of specific populations—such as cancer stem cells (CSCs) or drug‐resistant clones—that possess distinct methylome signatures. This technological advance is not merely an incremental improvement; it is a prerequisite for understanding core cancer hallmarks, such as the evasion of growth control and resistance to apoptosis, which are frequently governed by these specific subclones. This review specifically provides a comprehensive comparative overview evaluating the technical and chemical capabilities of emerging single‐cell modalities to distinguish DNA methylation profiles. By highlighting the strategic workflows of these emergent technologies alongside advanced computational tools, we emphasize how resolving these discrete cytosine variants empowers precise cell lineage tracing, the identification of refractory clones, and the implementation of locus‐specific epigenetic editing therapies against causal tumor subclones.

## Graphical abstract

Single‐cell DNA methylation (scDNAme) profiling maps epimutational clonal evolution, revealing mechanisms of malignancy and therapeutic resistance across diverse cancer types. By providing a high‐resolution landscape of intratumoral heterogeneity, these technologies empower precise patient stratification, guide the development of enhanced immunotherapies, and enable the design of locus‐specific epigenetic interventions for personalized oncology. Created in biorender. Kim, L. (2026) https://BioRender.com/eayb54c.

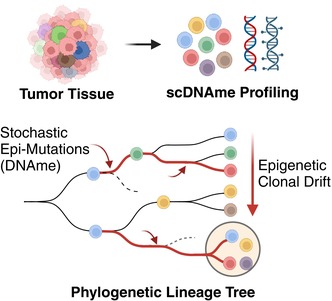

**Abbreviations**
5caC: 5‐carboxylcytosine
5fC: 5‐formylcytosine
5ghmC: 5‐glucosylhydroxymethylcytosine
5hmC: 5‐hydroxymethylcytosine
5mC: 5‐methylcytosine
AML: acute myeloid leukemia
CAR‐T: chimeric antigen receptor T‐cell
CD1A: cluster of differentiation 1A
CDKN2A: cyclin‐dependent kinase inhibitor 2A (p16)
cfDNA: cell‐free DNA
CLL: chronic lymphocytic leukemia
CNV: copy number variation
CRISPRa: CRISPR activation
CRISPRi: CRISPR interference
CTC: circulating tumor cell
dCas9: catalytically dead Cas9
FAM124B: family with sequence similarity 124 member B
FLT3‐ITD: FMS‐like tyrosine kinase 3 internal tandem duplication
IFI16: interferon gamma inducible protein 16
KRAS: Kirsten rat sarcoma viral (v‐ras) oncogene homolog
LAC: lung Adenocarcinoma
m6A: N6‐methyladenosine
NRAS: neuroblastoma RAS viral (v‐ras) oncogene homolog
PCA: principal component analysis
PCa: prostate cancer
RASA2: RAS p21 protein activator 2
SFN: Stratifin, also known as 14–3‐3 sigma
SNP: single nucleotide polymorphism
TME: tumor microenvironment

## 1 Introduction

DNA methylation, specifically the addition of a methyl group to the 5′ position of cytosine bases, is a key epigenetic regulatory process in development and cancer progression, governing essential functions such as cell specialization, embryogenesis, and genomic imprinting [1, 2, 3]. In the context of cancer, DNA methylation is a well‐studied epigenetic factor, offering promising potential as a cancer biomarker for diagnosis and prognosis [4, 5]. Aberrant DNA methylation patterns are universally observed across malignancies and contribute significantly to the disease phenotype [6].

Hypermethylation most commonly occurs at CpG Island (CGI) promoters and is generally correlated with reduced gene expression, leading to the epigenetic silencing of critical tumor suppressor genes (TSGs) [7, 8, 9, 10, 11]. This process is regulated by a coordinated epigenetic machinery in which DNMTs establish and maintain CpG methylation, UHRF1/2 reinforce methylation inheritance through replication‐coupled recruitment of DNMT1, TET enzymes counterbalance methylation via oxidative demethylation, and methyl‐CpG readers such as MeCP2 stabilize transcriptional repression [12, 13, 14, 15]. Consequently, the development of DNA methylation inhibitors is creating new therapeutic avenues [16].

On the other hand, global hypomethylation is frequently observed across the genome, particularly in intergenic and intragenic regions. In transcribed regions (gene bodies), methylation levels often positively correlate with gene expression [17]. Crucially, hypomethylation of enhancers—regulatory elements that dictate gene expression independent of promoter fluctuations—can reveal binding motifs for transcription factors (TFs), thereby inducing downstream expression changes of multiple target genes and promoting oncogenesis [18, 19]. Collectively, these disruptions in the methylation network, from global hypomethylation to the precise hypermethylation of TSG promoters, contribute directly to the initiation, progression, and metastatic capabilities of tumors by enabling malignant cells to bypass strict growth controls and resist programmed cell death.

Despite the importance of DNA methylation, traditional bulk sequencing methodologies, analyzing populations of thousands or millions of cells from a tissue sample, generate an average methylome profile, which obscures critical information about cellular heterogeneity [20, 21]. Cancer is characterized by profound heterogeneity—clonal, phenotypic, and spatial—involving malignant cells, various immune components, and stromal elements within the tumor microenvironment (TME) [22, 23, 24]. The limitations of bulk analysis mean that novel aberrant methylation patterns confined to rare subpopulations, such as early metastatic precursors or drug‐resistant clones, cannot be reliably detected or studied.

Single‐cell sequencing technologies, including scDNA sequencing, have fundamentally accelerated progress in oncology by enabling high‐resolution profiling of various malignant cell states, elucidating clonal adaptability and mechanisms of drug resistance [25, 26]. Single‐cell DNA methylation (scDNAme) analysis specifically allows researchers to explore previously inaccessible aspects of epigenetic heterogeneity associated with tumor biology, including clonal evolution, the molecular characteristics of cancer stem cells (CSCs) [27], the epigenetic contribution to treatment resistance [28], and the composition of circulating tumor cells (CTCs) [29]. This capacity to resolve heterogeneity at the single‐cell level is essential for developing therapeutic strategies that are individually tailored to specific cell types and targets. These reviews underline the increasing importance of single‐cell DNA methylation profiling for future clinical applications, providing a comprehensive understanding of precision oncology.

## 2 The technical evolution of single‐cell DNA methylation profiling

### 2.1 Advances in methylation chemistry and signal resolution

The technological evolution of scDNAme methods has been driven by the need to achieve reliable genome‐wide, single‐base resolution while maximizing throughput and minimizing technical artifacts. Many sequencing‐based approaches rely on chemical or enzymatic pretreatments to preserve methylation information prior to sequencing. A widely used method is bisulfite sequencing (BS‐seq), in which unmethylated cytosines are chemically converted to uracil (read as thymine), while methylated cytosines (5mC) remain protected [20]. However, bisulfite‐based single‐cell methylome profiling is limited by bisulfite‐induced DNA degradation, which substantially reduces library complexity and compromises input recovery. This catastrophic loss is particularly problematic for ultra‐low‐input materials like cfDNA, where preserving every molecule is critical for resolution. While affinity enrichment methods (e.g., methylated DNA immunoprecipitation (MeDIP) or methyl‐CpG‐binding domain sequencing (MBD‐seq)) are cost‐effective and widely used in bulk studies, they cannot achieve single‐base resolution and are limited in their ability to cover hypomethylated CpG sites [30].

Early technical optimizations centered on post‐bisulfite adapter tagging (PBAT), a strategy that integrates sequencing adapters after chemical conversion to ensure that even fragmented molecules are captured in the library [20, 31]. scWGBS (Single‐Cell Whole‐Genome Bisulfite Sequencing) utilizes tagged random hexamers for adapter integration [32]; while it is fast and cost‐effective, it often yields low library complexity due to redundant primer binding. In contrast, scPBAT specifically targets repetitive elements, offering reduced sequencing costs and PCR bias, which is particularly useful for tracking global hypomethylation—a hallmark of cancer instability—though it can suffer from low mappability in non‐repetitive regions [33]. snmC‐seq2 (Single Nucleus Methylcytosine Sequencing 2) further refines these approaches by isolating single nuclei, which avoids cytoplasmic contamination and improves the processing of solid tumor tissues [34]. While v1 of this method suffered from high levels of artifactual adapter dimer sequences, snmC‐seq2 incorporates a dephosphorylation step using Shrimp Alkaline Phosphatase (SAP) to inactivate carryover nucleotides, significantly improving library complexity and mapping rates.

**Fig. 1:**
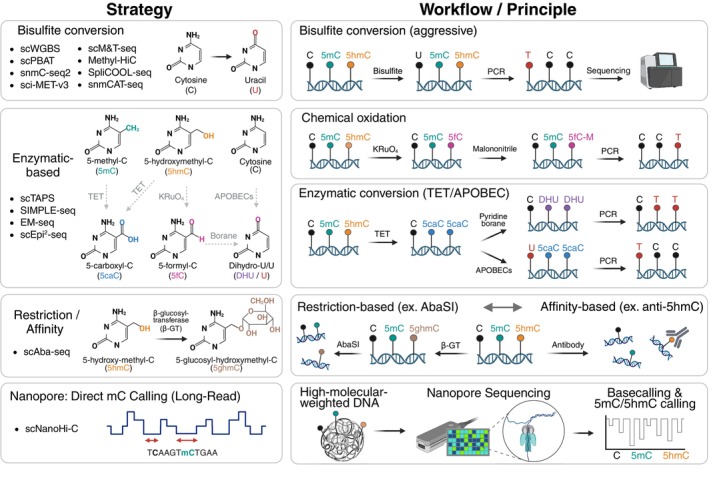
Single‐cell DNA methylation profiling technologies. The figure illustrates the chemical/enzymatic transformations (Strategy, left panels) and operational pipelines (Workflow/Principle, right panels) of the four primary categories of single‐cell genomic sequencing techniques utilized to resolve cytosine modifications, specifically 5‐methylcytosine (5mC) and 5‐hydroxymethylcytosine (5hmC). 5fC‐M, 5‐formylcytosine‐methylation; AbaSI, *AbaSI* restriction endonuclease; APOBECs, Apolipoprotein B mRNA editing catalytic subunits; DHU, Dihydrouracil; ONT, Oxford Nanopore Technologies; TET, Ten‐Eleven Translocation methylcytosine dioxygenases. The illustration was created with BioRender.com https://BioRender.com/01owyha.

Several specialized high‐resolution techniques have emerged from the PBAT framework, each offering distinct advantages for oncology research (Table 1). While PBAT substantially improved library efficiency, a notable recent advance has been the development of Enzymatic Methyl‐seq (EM‐seq) [35] (Fig. 1). By replacing chemical bisulfite conversion with a non‐destructive enzymatic workflow, EM‐seq uses TET2 to oxidize and protect methylated cytosines (5mC/5hmC) and APOBEC3A to deaminate unmodified cytosines, thereby preserving DNA integrity while improving library complexity and sensitivity. These advancements are particularly transformative for liquid biopsy applications, where the preservation of limited, highly fragmented cfDNA and circulating tumor DNA (ctDNA) is essential for longitudinal monitoring of the disease landscape [36]. A critical observation guiding these developments is that for resolving rare clonal populations in cancer, maximizing the number of cells profiled (throughput) is often more informative than maximizing depth per cell. To reach the scale necessary for tumor atlas construction, high‐throughput methods like sci‐MET (single‐cell combinatorial indexing for methylation analysis) prioritize scale over depth [37]. By using multiple rounds of barcoding (combinatorial indexing) on fixed nuclei, sci‐MET allows thousands of cells to be processed in pools, eliminating the need for expensive physical compartmentalization of individual cells.

**Table 1:** Comparative analysis of single‐cell DNA methylome sequencing methods. This table demonstrates scDNAme experimental platforms, categorizing them by their underlying molecular chemistry and cell‐capture strategies. It highlights the inherent experimental advantages (trade‐offs) between cell throughput (scale) and genomic CpG coverage per cell. Methods are additionally classified by their capability for multi‐modal co‐profiling and their optimal sample inputs. ATAC, assay for transposase‐accessible chromatin; Hi‐C, high‐throughput chromosome conformation capture; CNV, copy number variation; PBAT, post‐bisulfite adapter tagging; SNP, single nucleotide polymorphism; 5hmC, 5‐hydroxymethylcytosine; 5mC, 5‐methylcytosine.

Method	Core strategy	Key advantages	Modality	References
scWGBS	PBAT with tagged random hexamers	Fast and cost‐effective	DNAme	[32]
scPBAT	Repetitive element targeting	Low sequencing cost and reduced PCR bias	DNAme	[33]
snmC‐seq2	Single‐nucleus isolation and Adaptase integration	High library complexity; reduces cytoplasmic noise	DNAme	[34]
sci‐METv3	Combinatorial split‐pool indexing	High scalability without physical compartmentalization	DNAme	[37]
scAba‐seq	AbaSI nuclease‐dependent digestion	Single‐base resolution of 5hmC; enables 5hmC‐based lineage tracing	DNAme	[39]
EM‐seq	TET‐mediated oxidation of 5mC/5hmC + APOBEC3A to C	High DNA integrity and mapping efficiency; joint SNP/CNV calling.	DNAme + DNAhm (5mC/5hmC)	[35]
SIMPLE‐seq	Enzymatic glucosyl‐protection of 5hmC and deamination of C/5mC	High sensitivity for 5hmC; non‐destructive	DNAme + DNAhm (5mC/5hmC)	[40]
scTAPS	TET‐mediated oxidation of 5mC/5hmC to 5caC + borane reduction	High DNA integrity; high mapping rates and uniform coverage.	DNAme + DNAhm (5mC/5hmC)	[41]
scM&T‐seq	Physical separation of mRNA and gDNA within a single cell.	Direct linkage between transcriptional variation and distal methylation.	DNAme + RNA	[43]
snmCAT‐seq	T7‐based *in vitro* transcription, conversion for DNAme/ATAC.	Scalable Triple‐Omic resolution; high library complexity.	DNAme + ATAC + RNA	[47]
scEpi2‐seq	Capture of histone modifications (ChIP‐based/CUT&Tag‐like)	Resolves the interaction between DNAme and chromatin states	DNAme + Histone Marks (H3K27me3)	[48]
Methyl‐HiC	*In situ* Hi‐C and Bisulfite sequencing	Defines physical linkage between 3D loops and methylation alleles	DNAme + 3D Genome	[49]
SpliCOOL‐seq	Split‐pool ligation with universal Tn5 tagmentation	High‐throughput co‐profiling of DNA methylation and accessibility	DNAme + ATAC	[50]
scMHT‐seq	Strand‐specific enzymatic conversion + transcriptomic capture.	Combines lineage (5hmC) with function (RNA).	DNAme + DNAhm + RNA	[51]
scNanoHi‐C	Nanopore‐based sequencing of proximity‐ligated chromatin.	Resolves 3D contacts and DNAme across structural variants and repetitive regions.	DNAme + 3D Genome (Long‐Read)	[54]

For a more complete epigenetic picture, techniques must also distinguish between 5‐methylcytosine (5mC) and its derivative, 5‐hydroxymethylcytosine (5hmC) (Fig. 1). Oxidative Bisulfite Sequencing (oxBS‐seq) [38] and scAba‐seq [39] address this need, with scAba‐seq utilizing the AbaSI nuclease to detect 5hmC at single‐base resolution. Recent advancements have moved toward more integrated and non‐destructive methodologies. Single‐molecule Integrated Profiling of Long‐range Epigenetics (SIMPLE‐seq) [40] has emerged as a high‐throughput strategy for the joint detection of 5mC and 5hmC at single‐cell resolution, leveraging enzymatic conversion to avoid the pitfalls of traditional bisulfite treatments. Furthermore, the emergence of single‐cell TAPS (scTAPS: TET‐assisted pyridine borane sequencing) [41] represents a paradigm shift, enabling the direct catalytic conversion of modified cytosines without the destructive effects of bisulfite. This distinction is critical for lineage tracing and “epigenetic clock” applications, because 5hmC exhibits strand‐specific asymmetry during DNA replication, these technologies allow researchers to reconstruct precise cell‐division histories and track the longitudinal fate of individual clones [42].

### 2.2 Multi‐omics platforms for high‐resolution regulatory landscape

The evolution of single‐cell multi‐omics has fundamentally expanded the scope of oncology research by enabling the simultaneous capture of diverse molecular layers alongside DNA methylation, providing a high‐resolution view of the regulatory flow within complex tissues. Technical implementation of simultaneous multi‐omics relies on the precise isolation of genomic DNA and messenger RNA from a single cell to capture the direct relationship between stable epigenetic modifications and dynamic functional outputs. Methodologically, protocols like scM&T‐seq [43] (integrating scBS‐seq and Smart‐seq2) and Smart‐RRBS [44] utilize a complete lysis approach followed by the separation of mRNA using oligo‐dT‐coated magnetic beads. Alternatively, scMT‐seq [45] and scTrio‐seq [46] employ a gentle lysis strategy to physically partition the cell, allowing the cytosolic mRNA‐containing supernatant to be transferred to a separate tube while retaining the intact nucleus for genomic analysis. This physical separation is critical for performing bisulfite conversion on genomic DNA without compromising the transcriptome, facilitating the discovery of direct epigenome‐transcriptome associations—such as the inverse correlation between promoter methylation and gene expression—that are otherwise obscured by technical noise in unmatched datasets.

Foundational dual‐omics platforms like scM&T‐seq first enabled the parallel sequencing of the methylome and transcriptome, revealing the link between transcriptional variation and heterogeneous distal regulatory elements [43]. This was subsequently expanded by high‐throughput “Triple‐Omic” systems like snmCAT‐seq, which simultaneously capture the methylome, chromatin accessibility (ATAC), and transcriptome from the same individual nucleus [47]. Technical improvements such as scEpi2‐seq [48] utilize TAPS to provide simultaneous readouts of histone marks like H3K27me3 and CpG methylation, revealing how their interaction enforces gene silencing in treatment‐resistant populations. By integrating these layers with 3D structural data via methods like Methyl‐HiC, which connects chromatin conformation capture with bisulfite sequencing [49], researchers can now establish direct, unambiguous links between 3D loops and the methylation status of the enhancers they contact. Further scaling these insights, SpliCOOL‐seq utilizes split‐pool ligation to parallelly profile whole‐genome DNA methylation and chromatin accessibility in thousands of cells, successfully identifying distinct tumor subclones based on their coupled epigenetic‐structural landscapes [50]. Such integrated approaches are essential for resolving the “spatial paradox” of the tumor microenvironment, as they reveal how distal epigenetic architecture regulates gene expression programs driving phenotypic plasticity. Most recently, advancements like scMHT‐seq have integrated these multi‐modal layers with 5hmC resolution, offering a four‐dimensional analytical framework that combines cell state, 3D structure, and lineage history to track the longitudinal evolution of malignant clones [51].

Finally, direct sequencing platforms like oxford nanopore technologies (ONT) are transforming the field by detecting methylation marks through real‐time monitoring of electrical current as native DNA passes through protein nanopores. This method completely eliminates the need for DNA‐degrading bisulfite or enzymatic conversion, preserving the original genomic architecture and enabling the detection of diverse base modifications (5mC, 5hmC, m6A) in ultra‐long reads. Recent advancements have leveraged this capability to resolve the “dark matter” of the cancer genome to map methylation across repetitive elements and complex structural variants that are inaccessible to short‐read platforms [52]. Furthermore, the application of ONT to oncology has enabled the discovery of coordinated “epigenetic scars” on long DNA fragments, facilitating the identification of tumor‐specific haplotypes and allele‐specific methylation patterns in the tumor microenvironment [53]. Most notably, the integration of ONT with single‐cell platforms, such as scNanoHi‐C, now allows for the simultaneous profiling of 3D genome architecture and DNA methylation at kilobase‐to‐megabase scales, providing a transformative tool for tracking the clonal evolution of large‐scale genomic rearrangements [54].

## 3 Computational strategies for single‐cell DNA methylation analysis

### 3.1 Overcoming data sparsity and noise

The computational strategies for scDNAme analysis over the last decade have been defined by a transition from local statistical sharing to global, context‐aware modeling (Table 2). The primary challenge remains the extreme data sparsity and high technical noise, often compromising the clustering and interpretation of epigenetic profiles, as current sequencing techniques typically cover only a sparse fraction of the genome's CpG sites per cell. To compensate for the insufficient methylation values inherent to single‐cell measurements, methods frequently employ imputation, aggregating data from neighboring, similar cells to build “meta‐cells”. For instance, algorithms such as Melissa and scMET addressed this by utilizing hierarchical Bayesian frameworks, operating on borrowing statistical strength where the methylation state of a missing site is inferred by aggregating information from similar cells (clustering) or neighboring epigenomic features [55, 56]. This probabilistic approach ensures that unassayed sites are predicted based on the consensus of the cell's closest epigenetic neighbors, despite the sparse input.

**Table 2:** Computational strategies for single‐cell DNA methylation analysis. This table outlines bioinformatic algorithms for processing and interpreting sparse single‐cell methylome data. It classifies the tools by their primary algorithmic architecture and task focus: (1) quality control and imputation to overcome the challenges of extreme data sparsity; (2) lineage tracing tools that exploit stochastic, somatic “epimutations” as endogenous molecular clocks; and (3) multi‐modal integration frameworks designed to align sparse methylation profiles with transcriptomic data. [paired]: multimodality from the exact same cell, [unpaired]: multimodality from the different cells in a sample, [unmatched]: multimodality from the different cells across independent samples, [applicable]: applying for integration by general purpose. SNV, single nucleotide variation; RRBS, reduced‐representation bisulfite sequencing; TMRs, transcript‐proximal methylation‐associated regulatory sites; WCG, a specific trinucleotide sequence motif refers to either an ACG or a TCG sequence in the genome.

Method	Core algorithm	Strategy	Modality	References
**Overcoming data sparcity and noise**				
Melissa	Bayesian Hierarchical Model	Standalone clustering & local imputation	DNAme (WCG)	[55]
scMET	Hierarchical Bayesian Framework	Sharing information across cells/features to overcome sparsity	DNAme (WCG)	[56]
BPRMeth	Basis Function Regression	Spatial variation modeling (propensity functions)	DNAme (WCG), ATAC	[57]
scMeFormer	Transformer‐based Deep Learning	Captures long‐range genomic dependencies for imputation	DNAme (WCG)	[58]
scDNAm‐GPT	Mamba Backbone + Cross‐Attention	Foundation model; zero‐shot prediction of expression effects	DNAme (WCG), RNA	[59]
**Resolving clonal and lineage relationships**				
EPI‐clone	Epimutation‐based Tracing	Transgene‐free lineage tracing using stochastic epimutations	DNAme (Targeted), SNV, CNV	[60]
MethylTree	Shared CpG Similarity Matrix	Non‐invasive lineage reconstruction with signal de‐noising	DNAme (WCG or Targeted)	[61]
Sgootr	Distance‐based Phylogenetic	Noise‐minimizing site selection for metastatic histories	DNAme (WCG/RRBS)	[62]
**Integrating multi‐modalities**				
MAPLE	Supervised Ensemble Learning	Gene activity score modeling as a proxy for expression	DNAme (WCG)	[63]
scMD	Statistical Aggregation	Cell‐type signature matrix creation & deconvolution	DNAme (WCG)	[64]
MOFA+	Bayesian Matrix Factorization	[paired] Latent variable analysis to find shared axes of variation	DNAme, RNA, ATAC, Proteomics	[65]
scAI	Non‐negative Matrix Factorization	[paired] Manifold alignment and aggregation of sparse data	DNAme, RNA, ATAC	[66]
GLUE	Variational Autoencoder(VAE) + Guidance Graph	[unpaired] Prior knowledge‐driven manifold alignment	DNAme, RNA, ATAC	[67]
CCNMF	Coupled‐Clone Non‐negative Matrix Factorization(NMF)	[applicable] Maximizes concordance between expression and copy number	DNAme (CNV), RNA	[69]
INTEND	CCA‐based Joint Embedding	[unmatched] Identifies methylation‐associated regulatory sites (TMRs)	DNAme (WCG), RNA	[68]

Rather than treating sparse CpG sites as binary events, tools like BPRMeth model regional methylation as continuous “propensity functions” [57]. By capturing the spatial “shape” of a profile—such as the V‐shape at active promoters—this approach bypasses single‐cell noise and sparsity. These robust shape descriptors act as quantitative fingerprints, significantly improving cell clustering and gene expression prediction. The most recent shift involves the application of deep learning and large‐scale foundation models to capture long‐range genomic dependencies. scMeFormer uses a Transformer‐based architecture to achieve high‐fidelity imputation even when site coverage is reduced to as little as 10% [58]. Building on this, scDNAm‐GPT leverages state‐space models and bidirectional blocks to overcome the limited “window size” of previous models, effectively capturing dependencies across 20 million CpG sites and their surrounding nucleotide contexts [59]. By training on over 1 million cells, these models enable “zero‐shot” predictions, meaning they can interpret methylation dynamics in entirely new datasets without further training.

### 3.2 Resolving clonal and lineage relationships

A recent advancement, termed EPI‐clone [60], leverages targeted single‐cell measurements of DNA methylation at single‐CpG resolution to provide joint information on cellular differentiation states and clonal identities. The core algorithm principle behind EPI‐clone is the partition of CpG sites: one subset reflects functional differentiation, while another undergoes stochastic epimutations that serve as digital barcodes. This droplet‐based method allows for transgene‐free lineage tracing at scale, capturing hundreds of clonal trajectories across tens of thousands of cells without the complex genetic engineering required by traditional barcoding. Complementary strategy, MethylTree [61] operates on the principle of building cell–cell similarity matrices from jointly observed CpG sites, utilizing a mathematical regression to “de‐noise” the data by removing cell‐type‐specific signals that would otherwise confound lineage inference. Meanwhile, distance‐based phylogenetic tools such as Sgootr [62] utilize noise‐minimizing site selection to infer metastatic migration histories, allowing researchers to distinguish whether a tumor follows a sequential‐progression model or a more complex branched evolution. These computational advances enable the identification of aggressive, treatment‐resistant subclones and the real‐time monitoring of therapeutic resistance, transforming how clinicians interpret the evolutionary plasticity of the cancer ecosystem.

These computational advances also have profound implications for resolving the complexity of the cancer ecosystem. High‐resolution imputation and trajectory inference now allow researchers to distinguish rare, aggressive subpopulations like cancer stem cells and trace clonal evolution histories that were previously obscured. Furthermore, models like scDNAm‐GPT [59] enable reference‐free deconvolution of cell‐free DNA (cfDNA), facilitating the non‐invasive identification of tumor‐derived signals in liquid biopsies. This synergy between advanced algorithms and single‐cell resolution is transforming precision oncology by allowing for real‐time monitoring of therapeutic resistance and the discovery of novel epigenetic biomarkers for early cancer detection.

### 3.3 Computational integration of multi‐omics regulatory profiling

The clinical utility of simultaneous profiling methods is exemplified by scTrio‐seq, which concurrently analyzes the genome (copy number variations, CNVs), DNA methylome, and transcriptome within a single cell. By deducing CNVs directly from the single‐cell reduced representation bisulfite sequencing (scRRBS) data, this approach identified malignant subpopulations in human hepatocellular carcinoma (HCC) that were distinct across all three molecular layers, revealing how specific genomic rearrangements drive coordinated epigenetic and transcriptomic shifts. In studies where transcriptomic data is not directly available, computational frameworks such as MAPLE (Methylome Association by Predictive Linkage to Expression) [63] and scMD [64] bridge the gap by generating “gene activity scores”. These tools utilize supervised ensemble learning and statistical aggregation at the cluster level to model promoter and gene‐body methylation as proxies for gene expression. This proxy modeling enables researchers to identify cell‐type‐specific markers and perform reference‐free deconvolution in DNA‐only datasets, providing a high‐resolution view of the regulatory logic governing the cancer ecosystem.

The computational integration of single‐cell DNA methylation (scDNAm) and transcriptomic data relies on aligning disparate feature spaces. The most direct approach is paired integration, which utilizes paired multi‐omics assays (e.g., scNMT‐seq, scM&T‐seq) to profile multiple modalities within the exact same physical cell. Because the cell itself acts as a deterministic anchor, MOFA+ [65] generalizes Principal Component Analysis (PCA) to decompose paired matrices into shared latent factors, effectively isolating coordinated biological variation from assay‐specific noise. Similarly, scAI [66] employs an unsupervised iterative matrix factorization framework designed for sparse, near‐binary single‐cell epigenomics. It resolves sparsity by aggregating methylome signals across subgroups of similar cells identified through a co‐learned cell–cell similarity matrix. Conversely, unpaired integration maps different modalities from different cells of the same specimen. Lacking cell‐level anchors, methods like GLUE [67] rely on diagonal manifold alignment using a prior‐knowledge “guidance graph” with negative edge signs to capture the repressive relationship between gene‐body methylation and transcripts.

For complex designs, unmatched integration aligns disjoint modalities from different cells across independent cohorts. Frameworks like INTEND [68] resolve this disjoint bottleneck through cross‐modality translation, training a lasso model on bulk references (e.g., TCGA) to capture how methylation predicts expression before projecting single‐cell datasets into a unified space. Complementary general purpose clone mapping methods like CCNMF [69] align genomic and transcriptomic clones by maximizing global concordance between gene expression and copy number variation (CNV) profiles. This is highly applicable to scDNAm studies (e.g., scTrio‐seq) where subclonal CNVs are inferred directly from methylome sequencing coverage. Bridging these paradigms, foundation models like scDNAm‐GPT employ cross‐attention mechanisms over atlas‐scale pre‐trained datasets, enabling zero‐shot, reference‐free predictions of how specific cancer‐associated epigenetic alterations functionally rewire the single‐cell transcriptome.

## 4 Dissecting intratumoral heterogeneity by scDNA methylation

**Fig. 2:**
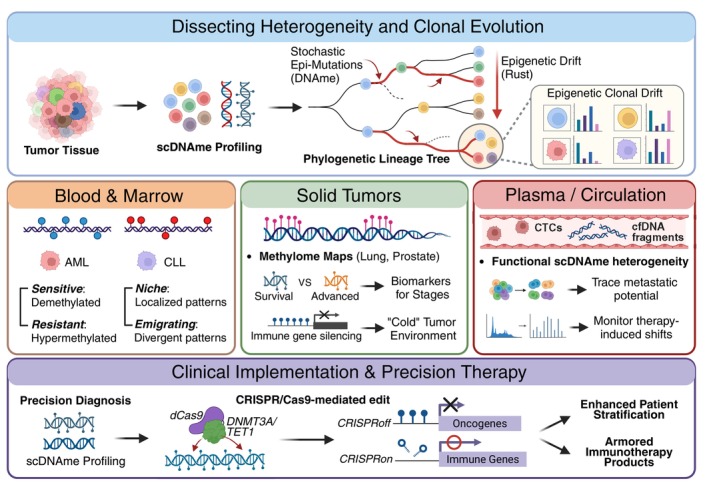
scDNAme dissects intratumoral heterogeneity and guides precision cancer therapy. The figure illustrates a multi‐stage framework utilizing scDNAme data. scDNAme profiling maps epimutational clonal evolution and quantifies rising transcriptional entropy. These specialized single‐cell techniques resolve distinct malignancy and resistance mechanisms across hematological malignancies (such as AML and CLL), solid tumors (including lung and prostate cancer), and liquid biopsies (evaluating CTCs and cfDNA). Ultimately, the resulting diagnostic datasets serve as a high‐resolution map for targeted interventions, enhancing patient stratification and guiding the design of armored immunotherapy products. AML, acute myeloid lymphoma, cfDNA, cell‐free DNA; CLL, lymphocytic leukemia, CTCs, circulating tumor cells. The illustration was created with BioRender.com. https://BioRender.com/y5zca1v.

scDNAme studies have begun to provide high‐resolution insights into the mechanisms of tumorigenesis across various tissue types, emphasizing how epigenetic landscapes shift to promote malignancy and resistance (Fig. 2).

In hematological malignancies, single‐cell resolution has become a prerequisite for resolving the intricate mutational and epigenetic landscapes that drive therapy resistance, evolutionary escape, and clinical relapse. In Acute Myeloid Leukemia (AML) and evolving myeloid neoplasms, innovative single‐cell methylomics platforms have exposed how intratumoral DNA methylation heterogeneity acts as a primary engine of drug resistance, moving beyond simple genetic tracking. Utilizing single‐cell transposable element methylation sequencing (scTEM‐seq) [70], researchers have shown that pronounced variance in global and region‐specific DNA methylation allows distinct leukemic subclones to actively resist hypomethylating agents (HMAs) like decitabine. Specifically, scTEM‐seq pinpoints discrete, resistant subclonal clusters that structurally block demethylation at high‐copy SINE Alu and LINE‐1 elements, leveraging this localized epiallelic stability to maintain the transcriptional silencing of critical immune‐process genes and evade host immunosurveillance. Furthermore, multi‐omic tracking via SCIMETAR‐seq has demonstrated that this pharmacological resistance is tightly coupled to cell‐state hierarchies within CD34+ hematopoietic stem and progenitor cells (HSPCs) transitioning toward secondary AML [71]. By mapping single‐cell DNA methylation simultaneously with somatic mutations and transcripts, SCIMETAR‐seq proved that while actively cycling leukemic cells are highly susceptible to HMA‐induced demethylation, quiescent leukemic stem cells (LSCs) maintain dense transposable element methylation—acting as an un‐reprogrammed, drug‐resistant reservoir that fuels post‐treatment disease progression.

Similarly, in Chronic Lymphocytic Leukemia (CLL), high‐resolution multiplexed single‐cell reduced‐representation bisulfite sequencing tracks stochastic methylation errors, termed epimutations [72, 73]. Operating as an internal molecular clock, these accumulated single‐cell epimutations allow researchers to reconstruct highly branched phylogenetic lineage trees, revealing that individual subclones progressively drift independently of genetic changes. Post‐treatment, these epiallelically unique cohorts preferentially emigrate from protective lymph node niches, directly linking specialized evolutionary trajectories to microenvironmental escape and survival against targeted small‐molecule inhibitors. By disentangling these subclonal dynamics, single‐cell approaches demonstrate that drug resistance is frequently an epigenetic phenomenon, providing a blueprint for multi‐targeted therapies designed to disrupt these protective epiallelic states before clinical relapse occurs.

The study of solid tumors has shifted toward resolving how the methylome signatures of the tumor microenvironment (TME) dictate disease progression and immune evasion. Lung adenocarcinoma (LAC) exhibits profound global methylation heterogeneity, which has been directly linked to advanced disease stages through single‐cell analysis [50]. Utilizing the high‐throughput SpliCOOL‐seq method, researchers have identified distinct lung cancer cell types based on parallel whole‐genome DNA methylation and chromatin accessibility profiling, identifying novel survival‐associated biomarkers such as *FAM124B* and *SFN*. In Prostate Cancer (PCa), single‐cell resolution has revealed that tumor cells utilize early epigenetic shifts to facilitate immune evasion prior to significant genetic alterations [11]. A hallmark discovery identified 40 core chromatin domains that are uniformly hypomethylated from the earliest stages of prostate malignancy through to metastasis. This hypomethylation specifically silences clusters of anti‐tumor immune genes, including the *CD1* gene cluster responsible for lipid antigen presentation and the *IFI16* innate immunity sensors, thereby creating a “cold” tumor environment that allows malignant cells to bypass immune surveillance.

Circulating Tumor Cells (CTCs) represent a critical diagnostic frontier where scDNAme profiling serves as a window into the metastatic cascade [74]. Because CTCs are rare and heterogeneous, bulk liquid biopsy methods often fail to capture their unique evolutionary trajectories. Single‐cell bisulfite sequencing (scBS‐seq) of individual CTCs has demonstrated that these cells possess distinct epigenetic signatures compared to primary tumor tissue, confirming that they represent evolutionarily divergent entities adapted for survival in the bloodstream [75]. These methylome profiles not only enable the tracing of diverse evolutionary histories but also serve as high‐resolution biomarkers for monitoring real‐time treatment response and predicting metastatic potential. Furthermore, the stability of DNA methylation allows for reference‐free deconvolution of cell‐free DNA (cfDNA), where models like scDNAm‐GPT can identify the specific tissue of origin for tumor‐derived fragments with high accuracy [59]. This capacity to resolve the “epigenetic ancestry” of circulating malignant material is essential for establishing non‐invasive longitudinal monitoring of tumor progression and therapy‐induced shifts.

From a different perspective, the resolution of “epigenetic aging” has introduced new paradigms for understanding tumor evolution. Single‐cell DNA methylation studies have mapped the concept of “epigenetic aging” or “drift,” identifying how stochastic errors in DNA methylation maintenance accumulate as “epigenetic rust” [76]. Advanced methods like scDEEP‐mC [77] allow for the observation of these dynamics during DNA replication, revealing that accelerated epigenetic age and increased entropy among tumor subclones are hallmarks of rapid proliferation and plastic cell states. By identifying these stochastic epiallelic shifts, researchers can now pinpoint the exact cells predisposed to malignant transformation and develop age‐aware diagnostic frameworks that distinguish deterministic oncogenic drivers from cumulative entropic damage.

## 5 Advancing clinical implementation and therapeutic strategies

Traditional epigenetic targeting has relied on broad inhibitors of DNA methyltransferases (DNMTs), such as 5‐azacytidine (5‐aza) and decitabine (DAC). These nucleoside analogues operate non‐specifically by targeting the entire epigenetic machinery, which can lead to the re‐expression of many tumor suppressor genes, but also induces unwanted global demethylation and toxicity. A critical paradigm shift is occurring with the emergence of CRISPR/Cas‐mediated macromolecular epigenetic editing. This technology utilizes catalytically inactive Cas9 (dCas9) fused to epigenetic effector domains (e.g., DNMTs or demethylases) to enable precise, locus‐specific manipulation of DNA methylation without inducing DNA double‐strand breaks.

This level of control offers unprecedented potential to reprogram the cancer epigenome. The true translational potential of scDNAme lies in its direct synergistic relationship with locus‐specific epigenetic editing. ScDNAme provides the high‐resolution diagnostic information necessary to inform precision therapy by resolving the map between a heritable epigenetic configuration and multiple transcriptional programs. By resolving the methylome of specific malignant subclones, scDNAme identifies the exact promoters or enhancers that have been aberrantly silenced or activated. For example, the CRISPRoff and CRISPRon systems utilize dCas9 fusions to either deposit methylation marks at target promoters for silencing or remove them for activation [78]. Researchers have demonstrated the efficacy of targeting p16 (CDKN2A) using dCas9‐DNMT to study cell proliferation or using CRISPRoff to silence the RASA2 gene in CAR‐T cells to create “armored” immunotherapy products that resist exhaustion [79]. Locus‐specific reprogramming provides a path toward overcoming the toxicity and global non‐specificity associated with traditional DNMT inhibitors. Similarly, single‐cell analysis in prostate cancer identified early hypermethylation at the CD1A‐IFI16 gene cluster, which silences lipid antigen presentation and innate immunity sensors [11]. This precise molecular lesion provides a clear blueprint for dCas9‐TET1‐mediated reactivation to restore anti‐tumor immunity. Integrating CRISPR‐based methylation reprogramming with precision oncology requires an understanding of which specific cell types need to be targeted. This strategy ensures that therapeutic edits are delivered effectively to aggressive, treatment‐resistant clones identified by scDNAme profiling while minimizing off‐target effects in surrounding healthy cells.

## 6 Conclusions and future perspectives

Single‐cell DNA methylation sequencing has fundamentally transformed the approach to cancer epigenetics by moving beyond the averaged methylome signal to resolve crucial cellular heterogeneity. This advancement has provided unprecedented insights into how epigenetic aberrations drive hallmark capabilities—such as clonal evolution, immune evasion, and drug resistance—in malignancies ranging from hematological cancers to solid tumors.

DNA methylation markers offer substantial promise for clinical oncology, providing superior sensitivity for early tumor screening and precise diagnosis compared to traditional biomarkers. The clinical utility of these markers is particularly evident in liquid biopsy applications, where the non‐invasive detection of methylation signatures in bodily fluids facilitates early‐stage screening and monitoring. Realizing this potential, however, necessitates a transition toward high‐sensitivity enzymatic conversion and direct nanopore sequencing to preserve the integrity of low‐input clinical templates. Despite these chemical advances, the translation of scDNAme profiling into routine clinical practice remains hindered by significant infrastructural and financial barriers. Overcoming the bottlenecks of high costs and limited sample accessibility requires the development of scalable, cost‐effective platforms alongside the integration of sophisticated Artificial Intelligence (AI). Foundation models such as scDNAm‐GPT are pivotal in this regard, providing the computational power to deconvolve cell‐free DNA (cfDNA) with the precision needed to pinpoint tissue‐of‐origin without the need for extensive reference maps.

The resolution of tumor evolution and cell lineage relationships is one of the most powerful applications of single‐cell epigenetics, as these dynamics are profoundly obscured in bulk analysis. While bulk tissue comparisons can only contrast the epigenome of a primary tumor site against a normal/peri‐tumor site or a metastatic site, single‐cell analysis can resolve the complete clonal evolution history of tumor cells, tracing the derivation and expansion of malignant populations. This algorithmic shift relies on the principle of treating stochastic “epimutations”—errors in DNA methylation maintenance—as a high‐resolution molecular clock. Because epimutations occur at a rate of approximately 0.001 per CpG site per division—orders of magnitude more frequent than somatic genetic mutations—they provide a rich, heritable signal that acts as a natural barcode for reconstructing a tumor's clonal and subclonal lineages [72].

As platforms evolve to optimize precision, the development of “5‐letter” and “6‐letter” sequencing platforms is eliminating the long‐standing sacrifice of canonical base accuracy for epigenetic detection [80]. These workflows simultaneously resolve all four genetic bases alongside 5mC and 5hmC, preserving phase information and enabling the detection of common C‐to‐T mutations—the most prevalent variants in cancer—which are typically obscured by traditional bisulfite conversion. The capacity to resolve 5hmC specifically through methods like scAba‐seq further provides an endogenous molecular clock, allowing for lineage reconstruction at individual‐cell‐division resolution without the need for synthetic barcodes [39]. These advancements are being synthesized into high‐resolution DNA methylation clocks and “epigenetic mitotic counters” that track the accumulation of stochastic errors, or “epigenetic rust,” identifying the specific cells predisposed to malignant transformation.

Collectively, these high‐resolution maps of the cancer epigenome provide the blueprint for the next generation of therapeutic strategies: the “rewiring” of the malignant state. The emergence of CRISPR‐mediated epigenetic editing (CRISPRi/CRISPRa) allows for the programmable, locus‐specific manipulation of DNA methylation to inhibit metastasis, sensitize tumors to immunotherapy, or induce the senescence of aggressive subclones. Transitioning from a precision oncology model focused on static mutations to one targeting dynamic regulatory logic enables the design of interventions that restore immune homeostasis or reverse the heritable epigenetic memory of drug resistance. Leveraging the depth and scalability of single‐cell DNA methylomics represents a pivotal transition toward achieving durable, personalized therapeutic outcomes through high‐resolution epigenetic precision.

## Conflict of interest

The author declares no conflict of interest.

## Author contributions

ISK contributed to conceptualization, writing, review & editing, visualization, and funding acquisition.
